# Viral Biomarkers for Hepatitis B Virus-Related Hepatocellular Carcinoma Occurrence and Recurrence

**DOI:** 10.3389/fmicb.2021.665201

**Published:** 2021-06-14

**Authors:** Yuanyuan Liu, Vaishnavi Veeraraghavan, Monica Pinkerton, Jianjun Fu, Mark W. Douglas, Jacob George, Thomas Tu

**Affiliations:** ^1^Department of Infectious Diseases, The Affiliated Xi’an Central Hospital of Xi’an Jiaotong University, Xi’an, China; ^2^Storr Liver Centre, The Westmead Institute for Medical Research, The University of Sydney and Westmead Hospital, Sydney, NSW, Australia; ^3^School of Medical Science, The University of Sydney, Camperdown, NSW, Australia; ^4^Marie Bashir Institute for Infectious Diseases and Biosecurity, University of Sydney, Sydney, NSW, Australia; ^5^Centre for Infectious Diseases and Microbiology, Westmead Hospital, Sydney, NSW, Australia

**Keywords:** hepatitis B, hepatocellular carcinoma, biomarkers, HBV surface antigen (HBsAg), HBV DNA integration, HBV RNA, HBcr antigen

## Abstract

Hepatocellular carcinoma (HCC) is the sixth most common cancer worldwide and the fourth leading cause of cancer-related death. The most common risk factor for developing HCC is chronic infection with hepatitis B virus (HBV). Early stages of HBV-related HCC (HBV-HCC) are generally asymptomatic. Moreover, while serum alpha-fetoprotein (AFP) and abdominal ultrasound are widely used to screen for HCC, they have poor sensitivity. Thus, HBV-HCC is frequently diagnosed at an advanced stage, in which there are limited treatment options and high mortality rates. Serum biomarkers with high sensitivity and specificity are crucial for earlier diagnosis of HCC and improving survival rates. As viral–host interactions are key determinants of pathogenesis, viral biomarkers may add greater diagnostic power for HCC than host biomarkers alone. In this review, we summarize recent research on using virus-derived biomarkers for predicting HCC occurrence and recurrence; including circulating viral DNA, RNA transcripts, and viral proteins. Combining these viral biomarkers with AFP and abdominal ultrasound could improve sensitivity and specificity of early diagnosis, increasing the survival of patients with HBV-HCC. In the future, as the mechanisms that drive HBV-HCC to become clearer, new biomarkers may be identified which can further improve early diagnosis of HBV-HCC.

## Introduction

Chronic infection with the Hepatitis B virus (HBV) is the predominant risk factor for primary liver cancer, specifically hepatocellular carcinoma (HCC; Bosch et al., 2004; Kew, 2010; Ozakyol, 2017). Overall, the lifetime incidence of HCC in HBV has been reported to be approximately 10–25% (McGlynn et al., 2015). Moreover, most cases of HBV-associated HCC occur in cirrhotic liver disease, present in 70–90% of cases (Yang et al., 2011). Liver cancer is the fourth most deadly cancer (Bray et al., 2018), with a median survival time as short as 11 months (Greten et al., 2005; Yang and Roberts, 2010). There is also a broad range of indirect health impacts driven by chronic HBV, including anxiety about disease progression, stigma and discrimination, and health care costs associated with treatment (Tu et al., 2020a).

Chronic HBV infection leads to a repeated cycle of liver damage and regeneration, which promotes tumorigenesis (Wang et al., 2006). Treatment of the underlying HBV infection can reduce, but not eliminate HCC risk (Papatheodoridis et al., 2015). Currently, oral nucleo(s/t)ide analogs (NAs) are used as first-line therapy for HBV infection. NA therapy targets the reverse transcriptase of HBV and suppresses HBV DNA replication, reduces progression to end-stage liver disease and improves long-term patient survival (Bitton Alaluf and Shlomai, 2016). NAs suppress viral replication (Ghany and Liang, 2007) but do not target HBV covalently closed circular DNA (cccDNA; Revill et al., 2020; Tu et al., 2020b). cccDNA is the template for HBV replication and expression of viral proteins, so its persistence plays a crucial role in chronic infection, inflammation, and cancer formation.

## Current Clinical Detection of HCC

Early screening of HBV-infected patients for HCC reduces mortality (Zhang B.H. et al., 2004). Current AASLD guidelines advise abdominal ultrasound surveillance for HBV-infected patients with advanced fibrosis or cirrhosis at 6-month intervals (Marrero et al., 2018), as marked liver fibrosis is a strong risk factor for HCC. However, ultrasound can miss HCC at early stages [sensitivity 63% (Singal et al., 2009)] and is strongly affected by operator- and patient-dependent factors (Singal et al., 2009; Pocha et al., 2013). Moreover, HCC can occur at any stage of liver fibrosis; hence AASLD guidelines recommend HCC surveillance of people with HBV who are ≥40 (for males) or ≥50 (for females) years old, regardless of fibrosis levels (particularly in those of Asian descent) (Xu et al., 2017). A cohort of studies suggested that liver stiffness measurement using FibroScan can predict HCC development in HBV patients with cirrhosis (Jung et al., 2011; Pesce et al., 2012; Adler et al., 2016) but fails to predict HCC in non-cirrhotic chronic hepatitis B (CHB) patients with liver stiffness measurement <8.0 kPa as well as patients with body mass index >28 kg/m^2^ and waist circumference ≥102 cm (Foucher et al., 2006; Jung et al., 2011; Cassinotto et al., 2016).

Alpha-fetoprotein (AFP) is the most widely used serum biomarker for the diagnosis of HCC (Marrero et al., 2018). However, elevated serum AFP is only found in 60–70% of HCC patients (Luo et al., 2019). Lectin-reactive AFP (AFP-L3) and des-gamma-carboxy prothrombin (DCP) have also been proven to be useful biomarkers for HCC (Li et al., 2001; Volk et al., 2007) and increase the sensitivity compared to using AFP alone (Marrero et al., 2009; Wang et al., 2020). Unfortunately, considering the low sensitivity (55%) of AFP-L3, HCC detection (particularly in early stages) is still suboptimal (Choi et al., 2019). DCP has a poorer diagnostic power for small HCC compared to AFP, but is better at detecting intermediate and advanced HCC (Nakamura et al., 2006).

Therefore, more sensitive, non-invasive biomarkers for better HCC diagnosis are needed. Here, we review the current knowledge on circulating viral biomarkers to screen for HCC, which may improve detection rates in combination with existing host-derived markers.

## HBV Structure, Natural History, and Replication Cycle

Hepatitis B virus is the prototypic member of the Hepadnaviridae family. The HBV virion contains a ∼3.2 kbp double-stranded DNA genome contained in a nucleocapsid composed of hepatitis B core antigen (HBcAg) subunits. The majority (∼90%) of virions contain a relaxed-circular DNA (rcDNA) genome, while a minority contain a double-stranded linear (dslDNA) form of the viral genome (Venkatakrishnan and Zlotnick, 2016). This nucleocapsid is enveloped in a host-derived lipid bilayer studded with hepatitis B surface antigens (HBsAg).

Infection with HBV itself is not cytopathic and the initial infection is usually asymptomatic, despite the production of high levels of virus antigen and viral particles by the liver. After decades of infection, HBV can trigger the immune response, though this is generally insufficient to clear all HBV-infected liver cells and subsequently causes chronic inflammation and liver damage progression. These two phases can be broadly divided serologically by the presence of circulating HBV e antigen (HBeAg, marking a status prior to extensive immune recognition) or antibodies against HBeAg (anti-HBe, present after antiviral clearance of the majority of infected cells). According to EASL 2017 Clinical Practice Guidelines (European Association for the Study of the Liver, 2017), chronic HBV infection can be separated into five clinical phases (Table 1): HBeAg-positive chronic HBV infection, previously termed “immune tolerant”; HBeAg-positive CHB with serum HBeAg positive, high HBV DNA and elevated ALT, termed “immune clearance phase”; HBeAg-negative chronic HBV infection, formerly known as the “inactive carrier” state; HBeAg-negative CHB with positive anti-HBe, persistent or fluctuating levels of HBV DNA and elevated ALT; HBsAg-negative phase, termed “occult HBV infection.”

**TABLE 1:** Natural history of patients with chronic HBV infection.

**Phases**	**New name**	**Old name**	**HBs**	**Anti-HBs**	**HBe**	**Anti-HBe**	**HBV DNA titers**	**ALT levels**	**Cirrhosis rate**	**HCC risk (incidence)^*a*^**
Phase 1	HBeAg-positive chronic infection	Immune tolerance	+	−	+	−	Very High	Normal	Very Low	0.04–0.5 (Fattovich et al., 2008)
Phase 2	HBeAg-positive chronic hepatitis	Immune active	+	−	+	−	High	Elevated	Low	0.5–3 (Chu et al., 2004; Lin S.M. et al., 2007; Fattovich et al., 2008)
Phase 3	HBeAg-negative chronic infection	Inactive carrier phase	+	−	−	+	Low to Undetectable	Normal	Low/Mid	0.02–0.2 (De Franchis et al., 1993; Hsu et al., 2002; Manno et al., 2004; Raffetti et al., 2016)
Phase 4	HBeAg-negative chronic hepatitis	Immune re-activation	+	−	−	+	Moderate to High	Elevated	Mid/High	No cirrhosis 0.3–0.6 (Fattovich et al., 2008)
										Cirrhosis 2.2–3.7 (Fattovich et al., 2008)
Phase 5	HBsAg-negative phase	Clearance or occult HBV infection	−	±	−	+	Undetectable to low	Normal	Low	No cirrhosis 0.3 (Kim et al., 2015)
										Cirrhosis 3 (Kim et al., 2015)

*^a^Incidence per 100 person years.*

**FIGURE 1:**
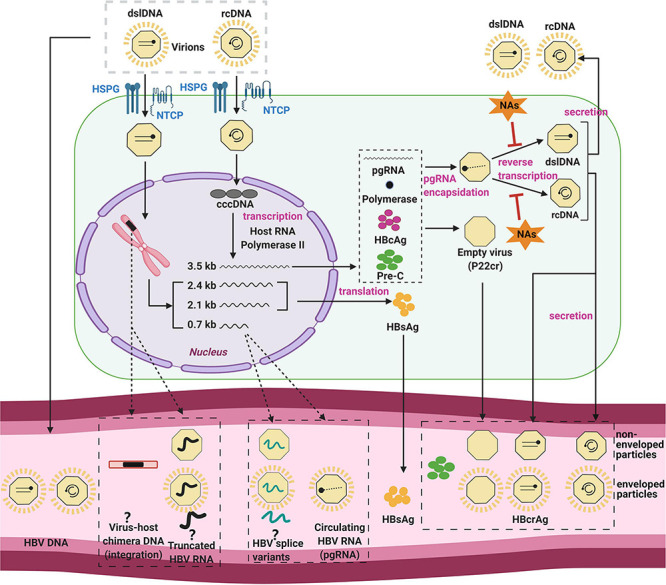
The HBV replication cycle and its secreted products. The HBV virion enters the hepatocyte by NTCP receptor binding, and uncoats prior to entry into the cytoplasm. The viral nucleocapsid is then transported to the nucleus, where it deposits its DNA genome. HBV relaxed-circular DNA (rcDNA) genomes can be repaired and ligated to form cccDNA, the template for all viral RNAs. HBV core antigen (HBcAg) is translated and forms capsids, some of which form around the pregenomic RNA (pgRNA) and viral polymerase. The pgRNA is reverse-transcribed to form either double stranded linear DNA (dslDNA) or rcDNA forms of the virus genome. The mature nucleocapsid is then enveloped by host membranes studded with HBV surface antigen (HBsAg) and secreted at multi-vesicular bodies. Cytoplasmic HBV capsids are recycled at a poor efficiency to the nucleus and do not appear to significantly add to the cccDNA pool (Tu and Urban, 2018; Revill et al., 2020; Tu et al., 2021). In a secondary pathway, HBV dslDNA can integrate into the host genome at host DNA breaks or form defective cccDNA (not shown). Some of these viral components are released in the serum (bottom) by as yet unclear mechanisms (dashed arrows) including within apoptotic bodies of dying hepatocytes, secretion through alternate pathways, or within exosomes. Even the form in which some of these biomarkers exist in the serum is still unknown and controversial (question marks). Figure was generated using Biorender (https://biorender.com/).

On a cellular level, the infection of hepatocytes begins with attachment of the virion to the sodium taurocholate co-transporting polypeptide (NTCP), the entry receptor of HBV (Yan et al., 2012, 2014; Ni et al., 2014; Figure 1). After binding and receptor-mediated endocytosis, viral nucleocapsids are transported through the cytoplasm (Yan et al., 2014) to the nuclear membrane, where uncoating and entry of the HBV DNA genome into the nucleus occurs.

Nuclear HBV rcDNA is converted into the HBV cccDNA form using host cell DNA repair enzymes (Königer et al., 2014; Qi et al., 2016). HBV cccDNA is an episomal “mini-chromosome” and acts as a stable template for the 5 viral mRNAs. Each of these transcripts has different 5′ transcription start sites but a common 3′ polyadenylation signal. These mRNAs include the 3.5-kb pregenomic RNA (pgRNA), the 3.5-kb precore RNA, the 2.4-kb/2.1-kb surface mRNAs, and the 0.7-kb X mRNA (Blondot et al., 2016). Moreover, post-transcriptional modification of 3.5-kb species can produce spliced HBV RNA variants (Candotti and Allain, 2017).

The functions of pgRNA are both as the template for reverse transcription and the translation of viral polymerase and core protein. The newly translated viral polymerase binds to the 5′-epsilon region of pgRNA, and is packaged together as sub-viral core particles (Jones and Hu, 2013). Reverse transcription occurs within the HBV nucleocapsid through a series of complicated mechanisms, resulting in the synthesis of rcDNA (major pathway) or dslDNA. These mature nucleocapsids are then enveloped by HBsAg and secreted into the blood at multi-vesicular bodies (Blondot et al., 2016).

Nuclear dslDNA genomes follow separate pathways: these can form replication-defective cccDNA (Yang and Summers, 1998) or integrate into the host cell genome (Yang and Summers, 1999; Tu et al., 2017). While the integrated HBV genome is replication-deficient, but still acts as a template viral antigen expression (e.g. HBsAg and HBx) (Wooddell et al., 2017).

A broad range of components generated by virus-infected cells have been investigated as potential biomarkers for predicting HCC occurrence (summarized in Table 2) and recurrence (summarized in Table 3). For each major serum viral marker that has been investigated, we provide in the next section a description, the mode of quantification, their molecular association with HCC, and their predictive power for HCC occurrence and recurrence.

**TABLE 2:** Serum viral biomarkers for the prediction of HCC occurrence.

**Biomarkers**		**Antiviral treatment**	**Patient population**	**Findings**	**References**
HBV DNA		Naïve	HBeAg (+)	HBV DNA was not different between HCC and non-HCC	Fung et al., 2007
			HBeAg (−)	HBV DNA is higher in HCC group (AUROC = 0.62)	Fung et al., 2007
			All patients	AUROC = 0.7	Tseng et al., 2012
		Treated	CHB patients	HBV DNA was not different between HCC and non-HCC	Kim et al., 2017; Lee et al., 2020
			Cirrhosis patients	Risk of HCC is significantly higher in low-level viremia (<2,000 IU/mL) compared to undetected	Kim et al., 2017
HBV integration		Naïve		Unreported	
		Treated		Unreported	
HBV variants	Splice variants	Naïve		Unreported	
		Treated	Severe fibrosis scores (F3/4)	Serum spliced HBV DNA with a cut-off value of 7% predicted HCC (AUROC = 0.77, sensitivity: 45%, specificity: 96%)	Bayliss et al., 2013
	Pre-S mutants	Naïve	HBeAg-negative patients without liver cirrhosis	HBV DNA with pre-S deletions predicted HCC (HR, 11.26; 95% CI, 2.18–58.1; *P* = 0.004), median time 84 months	Chen et al., 2007
		Treated	CHB patients with Genotypes C and B	HBV DNA with pre-S deletions predicted HCC (OR = 3.28).	Wungu et al., 2021
				HBV DNA with Pre-S1 or Pre-S2 mutations predicted HCC (OR = 2.42, 3.36)	Wungu et al., 2021
Total HBV RNA		Naïve		Unreported	
		Treated		Unreported	
Truncated HBV RNA		Naïve		Unreported	
		Treated		Unreported	
HBsAg		Naïve	HBeAg (−), HBV DNA > 2000 IU/mL	HBsAg poorly predicted HCC (AUROC: 0.58)	Tseng et al., 2012
			HBeAg (−), HBV DNA ≤2000 IU/mL	HBsAg ≥ 1,000 IU/mL is an independent risk factor for HCC (HR 13.7)	Tseng et al., 2012
		Treated		Unreported	
HBcrAg		Naïve	HBeAg (−), HBV DNA 2000–19,999 IU/mL	HBcrAg > 10,000 U/mL could independently define a high HCC risk group (HR 6.29)	Tseng et al., 2019
			HBeAg(−), HBV DNA≤10^4^ copies/mL, no cirrhosis	HBcrAg > 5012 U/mL was associated with HCC occurrence (HR 6.13)	Tada et al., 2016
			Any HBeAg status, HBV DNA > 10^4^ copies/mL, FIB-4 < 3.6	HBcrAg > 5012 U/mL was associated with HCC occurrence (HR 5.69)	Tada et al., 2016
			Independent of HBV DNA levels, HBeAg	HBcrAg > 794 U/mL was independently associated with HCC occurrence (HR 5.05)	Tada et al., 2016
		Treated	HBeAg (+)	HBcrAg > 4.9log U/mL predicted HCC (Sensitivity: 90.3%, specificity: 21.7%)	Hosaka et al., 2019
			HBeAg (−)	HBcrAg > 4.4log U/mL predicted HCC. (Sensitivity: 51.9%, specificity: 78.7%)	Hosaka et al., 2019
			HBV DNA (−) post-treatment	HBcrAg > 7.8 kU/mL predicted HCC., (AUROC: 0.61, Sensitivity: 57.9%, specificity: 70.4%)	Cheung et al., 2017
			Non-cirrhotic	HBcrAg > 7.8 kU/mL predicted HCC. (AUROC: 0.7, Sensitivity: 62.5%, specificity: 78.1%)	Cheung et al., 2017

**TABLE 3:** Serum viral biomarkers for the prediction of HCC recurrence.

**Biomarkers**		**Antiviral treatment**	**Patient population**	**Findings**	**References**
HBV DNA		Naïve	Early recurrence (within 2 years)	HBV DNA levels ≥20,000 IU/mL predicted microvascular invasion (HR 2.77; *P* < 0.001)	Sohn et al., 2014
			Late recurrence (after 2 years)	HBV DNA level >10^6^ copies/ml was associated with recurrence (HR 2.548, CI 1.040–6.240)	Wu et al., 2009
		Treated	1040 patients with a high baseline HBV DNA level (>2,000 IU/ml)	Undetectable HBV DNA at week 24 post-resection predicted lower late HCC recurrence (*P* < 0.001, HR 0.408, 95% CI 0.269–0.618), but was not associated with early HCC recurrence	Huang et al., 2013
HBV integration		Pre-resection: 21 (42.0%) Post-resection: 35 (70.0%)	50 HBV-related HCC with 36 genotype B (72.0%)	Detection of tumor-associated HBV DNA integrations in serum predicted HCC recurrence in >90% of cases	Li et al., 2020
HBV variants	Splice Variants			Unreported	
	Pre-S mutants	Naïve at HCC diagnosis: 35 (46%)	Median HBV DNA 2.1 × 10^4^ IU/mL	The AUROC of the pre-S2 plus pre-S1 + pre-S2 deletion percentage is 0.6827, followed by the combined pre-S deletion (AUROC,0.6789)	Teng et al., 2020b
		Naïve at HCC diagnosis: 35 (46%)	Median HBV DNA 2.1 × 10^4^ IU/mL	HBV DNA with Pre-S2 deletions (nt 1–54) in serum was associated with HCC recurrence (*P* = 0.008, AUROC = 0.6321)	Teng et al., 2020a
HBV RNA				Unreported	
Truncated HBV RNA				Unreported	
HBsAg		Naïve at HCC diagnosis: 202 (81%)	Late HCC recurrence (after 2 years)	HBsAg levels ≥ 4,000 IU/mL is the risk factor for HCC recurrence after 2 years (HR 2.80; *P* = 0.023)	Sohn et al., 2014
		Naïve at HCC diagnosis: 315 (78%)	Hepatic resection HBeAg(−) HBV DNA < 2000 IU/mL	HBsAg ≥ 1,000 IU/mL is associated with HCC recurrence	Huang et al., 2014
HBcrAg		Treated at diagnosis of HCC	55 HCC patients, either curative resection or percutaneous ablation	HBcrAg levels ≥ 4.8log U/ml at the time of HCC diagnosis was independent factor for HCC recurrence (HR 8.96, 95% CI 2.47–11.25; *P* = 0.005)	Hosaka et al., 2010
		Treated at diagnosis of HCC	119 HCC patients, HBeAg (−): 68%	HBcrAg level ≥ 5.1log U/ml was associated with increased tumor recurrence rate (*P* = 0.01)	Beudeker et al., 2021
		Treated at diagnosis of HCC	169 HCC patients with liver transplantation, HBeAg(+):47 (27.8%)	HBcrAg ≥ 5.0 log U/mL predicted HCC recurrence after 5 years (HR 5.27, 95% CI 2.47–11.25; *P* < 0.001)	Yu et al., 2019

## Serum Viral Biomarkers for HBV Related HCC

### HBV DNA

#### Description

Hepatitis B virus DNA, the genomic nucleic acid of the virus, reflects active viral replication and secretion. There are two forms of HBV genome: rcDNA and dslDNA (as mentioned in the HBV replication cycle) (Lee et al., 2004; Blondot et al., 2016). Quantitative PCR for serum HBV DNA detects both forms of the virus genome and is used as a clinical marker to measure the efficacy of antiviral therapy in people with CHB.

#### Quantification

Using real-time PCR quantification assays for HBV DNA detection is strongly recommended by EASL (European Association for the Study of the Liver, 2017) and is generally expressed as a WHO-standardized IU/mL (5.26 copies/mL = 1 IU/mL) (Saldanha et al., 2001). At present, with their high sensitivity, specificity, accuracy and broad dynamic range, these techniques are the most widely used assays in clinical practice. The assays include Cobas AmpliPrep/Cobas TaqMan HBV version 2.0 (CAP/CTM HBV 2.0) (Roche Molecular Systems, Pleasanton, CA, United States), with a dynamic range between 10^5^ copies/mL to 9 × 10^8^ copies/mL, and Abbott RealTime HBV assay (Abbott Molecular, Des Plaines, IL, United States), with a dynamic range 50 copies/mL to 5 × 10^9^ copies/mL (Chevaliez et al., 2008, 2010; Yeh et al., 2014). More sensitive pre-clinical tests have also been developed: digital droplet PCR can quantify HBV DNA down to 8 copies/mL (Liu et al., 2017).

The amount of the dslDNA form of HBV (as opposed to rcDNA form) can be measured using quantitative real-time PCR (qPCR) coupled with peptide nucleic acid-mediated clamping (Zhao X.L. et al., 2016). However, this assay is not a standard laboratory test.

#### Molecular Association With HCC

Serum HBV DNA load in people with CHB has been shown to be closely related to disease activity and progression (Iloeje et al., 2006). Moreover, elevated HBV DNA is considered as a predictive biomarker for HCC, independent of HBeAg and liver cirrhosis (Chen et al., 2006, 2011). HBV DNA is associated with both indirect and direct mechanisms of carcinogenesis. The indirect mechanisms include inducing new HBV infection of hepatocytes, which triggers ongoing liver immune attack, inflammation, and liver injury (Bolukbas et al., 2005; Duygu et al., 2012; Chen and Tian, 2019). Possible mechanisms of direct carcinogenesis include HBV dslDNA integration into the host genome, which reportedly leads to genomic instability, insertional mutagenesis and expression of pro-oncogenic viral proteins (Sze et al., 2013; Zhao L.H. et al., 2016; Gao et al., 2019). Indeed, one study reported that the levels of dslDNA increased to 14% of total serum HBV DNA in people with liver cirrhosis and 20% in those with HCC, compared to 7% in people with CHB alone (Zhao X.L. et al., 2016). However, the utility of dslDNA proportion as a biomarker for HCC has not been examined in clinical trials.

#### Performance as a Predictor of HCC

##### HCC occurrence

In NA-naïve patients, two studies in Taiwan have inferred that elevated serum HBV DNA level can be a useful biomarker for monitoring HCC independent of HBeAg and liver cirrhosis (Chen et al., 2006, 2011). A study by Chen et al. (2011) showed that in a cohort of patients with genotype B/C HBV infection aged >30 years, the risk of HCC increased with higher levels of circulating HBV DNA (after excluding patients in immune tolerance phase with HBV DNA >10^7^ copies/mL, as people in this phase have low risk of HCC). In a case-control study of HBeAg-negative CHB patients, levels of HBV DNA were found to be higher in people with HCC than those without (Area Under the Receiver Operating Characteristic curve, AUROC = 0.62) (Fung et al., 2007). Tseng et al. (2012) reported that in a cohort of 2688 treatment-naïve people with CHB, HBV DNA predicts the risk of HCC regardless of HBeAg status [AUROC = 0.7(95% confidence interval (CI): 0.65–0.75)]. Together, this suggests HBV DNA has good predictive strength for HCC risk.

However, HBV DNA titers cannot be used for all patients. NA therapy can reduce levels of serum HBV DNA to an undetectable level, preventing its use as a biomarker in this population that is still susceptible to HCC (Vlachogiannakos and Papatheodoridis, 2013; Varbobitis and Papatheodoridis, 2016). Further, in a Korean cohort of 1,246 patients with CHB who received entecavir, baseline HBV DNA did not predict HCC in non-cirrhotic patients under NA treatment (>5.7 vs. <5.7log IU/mL; *P* = 0.166) (Kang et al., 2017).

Nevertheless, HBV DNA levels can be used to detect poor response to NAs, which is linked to HCC. In a cohort of 875 patients with CHB treated with entecavir, greater HBV DNA levels were linked to increased HCC risk in patients with cirrhosis (adjusted hazard ratio = 2.20, compared to those with persistently undetectable HBV DNA) (Kim et al., 2017). But, HBV DNA did not predict HCC risk in patients without cirrhosis. HCC incidence was not significantly different between people with persistently detectable HBV DNA and those with undetectable levels (13.3% vs. 8.3%, *P* = 0.821) (Lee et al., 2020). Thus, in patients treated with NAs, HBV DNA titer is useful in predicting HCC only in cirrhotic patients.

##### HCC recurrence

In NA-naïve HCC patients, high serum HBV DNA levels were an independent risk factor for HCC recurrence after curative resection or liver transplantation, or percutaneous radiofrequency ablation (Huang et al., 2008; Chuma et al., 2009; Goto et al., 2011; Li et al., 2011). In a study of 248 Korean patients who underwent curative resection for early stage HBV-related HCC, HBV DNA level ≥20,000 IU/mL [hazard ratio (HR) 2.77; *P* < 0.001] was a risk factor for microvascular invasion and early recurrence (within 2 years) (Sohn et al., 2014). However, Wu et al. (2009) found that HBV DNA level >10^6^ copies/mL (HR 2.548, CI 1.040–6.240) in Taiwan patients with HBV-related HCC was associated with late recurrence (after 2 years). Therefore, the utility of high HBV DNA in predicting HCC recurrence needs further research.

After NA treatment at diagnosis of HCC or follow-up, sustained HBV DNA expression could increase the risk of HCC recurrence (Kim et al., 2008). Moreover, Huang et al. (2013) found that in the 865 HCC patients receiving NAs therapy with a high baseline HBV DNA level (subpopulation of a 1,040 patient cohort), an undetectable HBV DNA before postoperative week 24 (*P* < 0.001, HR 0.408, 95% CI 0.269–0.618) was a protective factor for late HCC recurrence, but not for early tumor recurrence (*P* = 0.541, HR 0.946, 95% CI 0.793–1.130). Therefore, detectable HBV DNA level could predict HCC recurrence in patients receiving NA treatment.

### HBV Integration

#### Description

Integration of the dslDNA form of HBV DNA can occur throughout the host genome at double-strand DNA breaks (Bill and Summers, 2004), likely without the help of specific viral proteins (Tu et al., 2019) (instead probably using host DNA repair enzymes). The sites of HBV DNA integration during CHB are randomly distributed across the host genome without strong preference for any specific structural genome features (Budzinska et al., 2018a).

#### Quantification

Hepatitis B virus integrations can be detected in the serum and tissue of HBV-infected patients as virus-host chimera DNA. Current detection methods for virus-host chimera DNA include whole-exome sequencing, whole-genome sequencing, *Alu* PCR and inverse-nested PCR (Budzinska et al., 2018b). These have shown less-than-genome length fragments of HBV dslDNA integrate (with terminal truncations of 100s to 1,000s of base pairs being common). Of these detection assays, the only method enabling absolute quantification of HBV integrations is inverse-nested PCR (Mason et al., 2009), though this method is very time-consuming and technically challenging, limiting its clinical utility.

#### Molecular Association With HCC

While HBV integration sites are randomly distributed across the genome in non-tumor tissue, HBV DNA integrations in HCCs have been reported to be enriched in genes involved in carcinogenesis pathways (i.e., *CTNNA2, EGFR*, and *TERT*) and have been found to be preferentially located within CpG islands and close to telomeres (Sung et al., 2012; Zhao L.H. et al., 2016; Li et al., 2019). Even when the HBV infection is cleared (marked by HBsAg seroconversion), HCCs risk remains and 70% of HCCs contain HBV integrations (Wong et al., 2020).

The mechanism behind the association of HCC with HBV integration is currently unknown. Many studies indicate that HBV integration causes genetic damage and chromosomal instability, which has the potential to promote carcinogenic transformation (Scotto et al., 1983; Zhao L.H. et al., 2016; Chen et al., 2019; Jang et al., 2020), or drive downstream host protein expression. HBV DNA can integrate into fragile sites, CpG islands and near telomerase reverse transcriptase, lysine methyltransferase 2B, as well as cyclin A2 (Wong et al., 2020), potentially inducing cancer-initiating genomic instability (Zhao L.H. et al., 2016; Furuta et al., 2018; Wong et al., 2020). However, genomic instability is not evident in many cases of HBV-HCC (Sung et al., 2012). The integrated HBV DNA can also disrupt cellular genes by insertional mutagenesis or drive expression of nearby with viral promotors. Insertion in TERT promoter, CCNE1 (cyclin E1), CCNA2, MLL4 (Myeloid/lymphoid or mixed-lineage leukemia 4), TP53, and CTNNB1 have been repeatedly detected in HCC (Paterlini-Brechot et al., 2003; Sung et al., 2012; Kawai-Kitahata et al., 2016), but these are not present in all tumors.

In addition, mutant HBsAg produced from integrated HBV DNA could contribute to HBV-related HCC by causing endoplasmic reticulum (ER) stress and immune evasion (Hsieh et al., 2004; Wang et al., 2006).

#### Performance as a Predictor of HCC

##### HCC occurrence

Specific HBV-host fusion genes created by HBV integrations have been suggested as biomarkers for predicting HCC in people with CHB. In NA-treated patients, a prospective study using liver tissue from people with CHB reported that human ESPL1-HBV S fusion gene was detected in 8 of 12 (66.7%) people with HCC, compared to 0 of 11 (0%) CHB patients without HCC (Hu et al., 2020). Moreover, HBV has been reported to integrate into long interspersed nuclear elements (LINEs), leading to fusion HBx-LINE1 transcripts. HBx-LINE1 can activate β-catenin signaling, reduce E-cadherin and enhance cell migration, which has been suggested to promote HCC progression (Liang et al., 2016). These studies suggest that specific fusion genes could be used as a biomarker for the early detection of HCC in people with CHB, but these have not been able to be repeated independently in other cohorts [for example, in a cohort of Vietnamese patients with HBV-associated HCC (Trung et al., 2019)]. Indeed, the majority of integration sites in tumor samples are randomly distributed across the host genome (Zhao L.H. et al., 2016).

##### HCC recurrence

Several studies have found that circulating viral-host chimeric DNA (vh-DNA) generated from HBV integration may be a useful biomarker for monitoring HCC recurrence (Wang et al., 2019; Li et al., 2020). A study of 20 people with HBV-HCC found circulating vh-DNA representing 87 different HBV integration sites, which were enriched in genes involved in cancer-related pathways, suggesting they could act as a biomarker for HCC diagnosis (Li et al., 2019). Moreover, Li et al. (2020) detected vh-DNA in 97.7% of people with HBV-related HCC. Two months following HCC resection, the same vh-DNA sequence could be detected in 10 cases (23.3%), nine of whom (90%) experienced HCC recurrence within a year. Thus, vh-DNA of HBV integration could also be a useful circulating biomarker for monitoring HCC recurrence.

### HBV Splice Variants

#### Description

Hepatitis B virus pgRNA has multiple splice donor and acceptor sites and can be spliced by cellular machinery as a post-transcriptional modification. Sixteen spliced pgRNA variants have been identified both *in vitro* and in tissues of CHB patients (Candotti and Allain, 2017). These splice variants can be encapsidated, reverse-transcribed and secreted into serum as replication-deficient viral particles (Terre et al., 1991).

Spliced viral RNAs can also be translated into HBV spliced proteins. For example, the 2.2-kb singly spliced variant lacking intron 2447/489, can encode hepatitis B spliced protein (HBSP) in the livers of patients with chronic HBV infection (Soussan et al., 2000). The 2447-2901 HBV RNA splice variant can act as the template for a 43 kDa polymerase-surface fusion glycoprotein (P-S FP), which localizes to the ER and is posited to be an HBV structural protein (Huang et al., 2000; Park et al., 2008).

Furthermore, hepatitis B doubly spliced protein and HBSP are respectively encoded by the 2.2-kb doubly spliced pgRNA and the single spliced product 1(SP1) variant (Terre et al., 1991; Huang et al., 2000; Soussan et al., 2000; Lee et al., 2008). However, the specific function of any of these splice variant-derived proteins is currently unclear.

#### Quantification

Hepatitis B virus splice variants can be quantified by reverse-transcription PCR (RT-PCR). At present, using different combinations of 5′ splice site and 3′ splice site can generate HBV RNA splicing variants, including 16 identified HBV splice variants of pgRNA and 4 splice variants of preS2/S mRNA (Su et al., 1989; Terre et al., 1991; Hass et al., 2005). The 2.2-kb singly spliced variant with a lack of intron 2447/489 which is the most common spliced variant can encode the HBSP, which can be detected by Western blot (Soussan et al., 2003).

#### Molecular Association With HCC

Some studies have reported increased HBV RNA splicing being associated with HCC (Kremsdorf et al., 2006; Bayliss et al., 2013). The 2.2 kb HBV spliced variant has been reported to be more highly expressed in tumor tissues than in the adjacent-tumor tissues (Lin et al., 2002). Moreover, when full-length (3.2 kb) HBV DNA and 2.2 kb spliced variant are co-transfected into HepG2 cells, the replication signal of the 3.2 kb HBV genome was increased 3–7 times (Lin et al., 2002). This suggests the HBV spliced variant plays a role in increasing HBV, which is a strong risk factor for HCC.

Circulating splice variant DNA is most frequently detected as defective HBV particles (dHBV) derived from reverse transcription of the 2.2-kb singly spliced mRNA, the most common spliced variant (Günther et al., 1997). In NA-naïve patients, the ratio of serum dHBV to wild-type HBV was lower in patients with moderate fibrosis and moderate or no liver necrosis compared to those with severe fibrosis and severe liver necrosis (Soussan et al., 2008). However, the direct clinical relationship between HBV splice variants and HCC remains uncharacterized.

#### Performance as a Predictor of HCC

##### HCC occurrence

Many studies have suggested that Pre-S deletion mutants play an important role in HBV-related HCC (Chen et al., 2007; Xie et al., 2010; An et al., 2018; Chen, 2018). In NA-naïve patients, a study enrolled 141 HBeAg-negative patients with CHB, 7 of whom developed HCC with a median time of 84 months. Univariate analysis showed that the presence of pre-S deletions was a significant factor for prediction of HCC (HR 11.26, 95% CI, 2.18-58.1; *P* = 0.004) (Chen et al., 2007). A recent meta-analysis revealed that pre-S deletions were related to HCC occurrence (OR 3.28, 95% CI 2.32–4.65; *P* < 0.00001; random-effects model). Both pre-S1 and pre-S2 were risk factors for HCC development, with OR 2.42 (95% CI 1.25–4.68, *P* = 0.008) and 3.36 (95% CI 2.04–5.55; *P* < 0.00001), respectively (Wungu et al., 2021).

In a cohort of 165 people with CHB under NA treatment in Australia (58 of whom were diagnosed with HCC), the median level of serum spliced HBV was higher in HCC patients than in non-HCC patients (*P* < 0.001) (Bayliss et al., 2013). Using a real-time PCR cut-off value of 7% for serum spliced HBV, the AUROC analysis of spliced HBV is 0.77, with a sensitivity of 45% and a specificity of 96%. Multiple regression analysis found that the serum spliced HBV level increased by about 0.1% per year before the diagnosis of HCC, independent of liver fibrosis (Bayliss et al., 2013).

##### HCC recurrence

Studies have revealed that HBV-related HCC patients with pre-S mutants are at higher risk of HCC recurrence after curative surgery, even when receiving post-surgical NA therapy (Su et al., 2013; Yen et al., 2018; Teng et al., 2020b). Su et al. (2013) analyzed 73 HCC patients without NAs therapy but with pre-S deletion mutants. They found that pre-S deletion mutants were related to a higher rate of HCC recurrence and higher serum HBV DNA levels (*P* = 0.055) (Su et al., 2013). Moreover, a recent study reported that using next-generation sequencing-based quantitative detection of pre-S mutants in serum can be useful for predicting HCC recurrence (AUROC of either pre-S2/pre-S1 or pre-S2 deletion = 0.683) (Teng et al., 2020b). Teng et al. (2020a) reported that only the presence of pre-S2 deletions (nt 1 to 54) in serum was associated with HCC recurrence (*P* value = 0.0080) with higher AUROC (0.632, 95% CI 0.556–0.708), compared with the pre-S1 deletion or the pre-S1 + pre-S2 deletion (nt 2,855–2,872, 1–54). In summary, pre-S2 deletion mutants may be a useful biomarker for HCC recurrence.

### Circulating HBV RNA

#### Description

Multiple studies have shown that HBV RNA can be detected both in culture supernatants and in the serum of people with CHB (Hatakeyama et al., 2007; Huang et al., 2015; Wang et al., 2016). Given that HBV RNA exists as pgRNA in virus-like particles [produced by defective or partial reverse transcription (Wang et al., 2016; Prakash et al., 2018)], theoretically, serum HBV RNA is derived only from cccDNA in infected hepatocytes. However, the mechanism of the release of HBV pgRNA viral particles from infected hepatocytes into the circulation is unclear (Lam et al., 2017; Butler et al., 2018; Wang et al., 2018).

#### Quantification

Serum HBV RNA can be measured by quantitative RT-PCR, and digital droplet PCR (Wang et al., 2016; van Campenhout et al., 2018; Carey et al., 2019). Butler et al. (2018) used quantitative RT-PCR on the m2000 system (Abbott Molecular) to quantify serum HBV RNA detection with a lower limit of quantitation of 45 U/mL (Carey et al., 2020). There is limited standardization between these approaches to HBV RNA quantification, so further work needs to be done to harmonize these assays if they are to be used for routine diagnosis.

#### Molecular Association With HCC

Serum HBV RNA is closely related to the activity of HBV replication, especially in people with CHB during NA treatment (Giersch et al., 2017; Lu et al., 2017; Wang et al., 2017; Huang et al., 2018). However, there are few data on its predictive power for HCC risk. Halgand et al. reported that HBV pgRNA levels in tumor tissues were correlated with a particular HCC subtype (well-differentiated, non-invasive, and associated with better survival) (Halgand et al., 2018). However, serum levels may not be correlated with this. It is possible that high levels of HBV RNA could be a predictor of HCC in people with CHB under NA treatment (given it is a surrogate of cccDNA activity), but there is no clear clinical evidence for this yet.

### HBV Truncated RNA

#### Description

Hepatitis B virus integration can act as a template for truncated HBV RNA (trRNA) transcripts. Hilger et al. (1991) identified HBV trRNA transcripts that terminated at a non-canonical CATAAA polyadenylation signal within the 3′ end region of the HBx open reading frame in tissue samples from two HBV-HCC patients. This signal can be used when the canonical polyadenylation signal is absent (e.g., when truncated as in the integrated HBV DNA form) (Breitkreutz et al., 2001). Later studies suggested that truncated HBx transcripts with a C-terminal deletion could be transcribed from integrated HBV DNA (Wang et al., 2004).

#### Quantification

Using specific primers containing a sequence corresponding to the polyadenylated 3′-end of full-length polyadenylated HBV RNA (flRNA) or trRNA, RACE-PCR targets the 3′-ends of the X gene for quantification of all polyadenylated HBV RNA species (Zhang W. et al., 2004; Ou et al., 2020). The assay’s lower limit of detection for HBV RNA was 794 copies/mL with a quantitative range of 800–10^6^ copies/mL.

### Molecular Association With HCC

Studies have shown that HBV trRNA, which can be transcribed from integrated HBV DNA, can encode a C-terminal truncated HBx protein (Hilger et al., 1991; Sze et al., 2013; van Bömmel et al., 2015). C-terminal-truncated HBx has been reported to enhance HCC invasion and reduce apoptotic response (Tu et al., 2001; Ma et al., 2008). *In vitro* studies suggest that C-terminal-truncated HBx promotes HCC through upregulating caveolin-1 to enhance β-catenin-mediated transcription of FRMD5 (FERM domain containing 5) (Ng et al., 2016; Mao et al., 2019). Sze et al. (2013) analyzed clinical data from 50 HBV-HCC patients and found that C-truncated HBx correlated with venous invasion. Also *in vitro* experiments reported that C-truncated HBx activates matrix metalloproteinase 10 by increasing C-Jun transcriptional activity, resulting in enhanced cell invasion and metastasis (Sze et al., 2013). Moreover, C-terminally truncated middle surface protein MHBst initiates c-Raf-1/Erk-2 signaling, resulting in an increased hepatocyte proliferation rate and dysplastic changes in hepatocytes (Hildt et al., 2002; Wang et al., 2006). Although these suggest possible roles for truncated HBV protein in tumor progression, whether it also plays a role in tumor formation is not clear.

#### Performance as a Predictor of HCC

Although serum HBV trRNA has been detected and used as a predictor for virological outcomes (van Bömmel et al., 2015), its association with HCC has only been shown in tumor tissues and not serum. A study with 50 people with HCC revealed that C-terminal truncated HBx was detected in 23 of 50 (46%) tumor tissues, and these had more venous invasion compared to tumors expressing only full-length HBx (*P* = 0.005) (Sze et al., 2013). This is consistent with another study where C-terminal truncated HBx was detected in 88 of 111 (79.3%) HCC tissues, compared with full-length HBx in all 111 non-tumor tissues and 23 of 111 (20.7%) HCC tissues (Ma et al., 2008). However, these studies only detected truncated HBx in confirmed HCC tissues. Whether serum HBV trRNA can predict HCC occurrence or recurrence is still unclear.

### Hepatitis B Surface Antigen

#### Description

Hepatitis B virus sub-genomic mRNA transcripts (2.4- and 2.1-kb surface mRNAs) encode the large, middle, and small variants of the HBV surface antigen, which can assemble at the ER as sub-viral particles (SVP) and be secreted via the Golgi apparatus (Ganem and Schneider, 2001). The majority of circulating HBsAg exists as non-infectious filamentous and spherical SVP, in 1,000- to 100,000-fold excess compared to virions (Wei et al., 2010). HBsAg may be translated from both HBV cccDNA and integrated DNA; the latter especially in HBeAg-negative chronic HBV-infected patients (Hu et al., 2018).

Secreted HBsAg in SVP could play an immunomodulatory role during HBV infection. SVP capture neutralizing anti-HBsAg antibodies and divert host immune recognition away from infectious HBV virions (Carman et al., 1996). The host antiviral immune response is subsequently minimized, allowing HBV persistence (Rydell et al., 2017). HBsAg is also the target of HBV functional cure; if serum HBsAg is eliminated then HBV infection is considered to be cleared (Chen et al., 2016; Al Awaidy and Ezzikouri, 2020).

#### Quantification

Currently, there are three quantitative assays for HBsAg measurement: Architect HBsAg QT (Abbott Diagnostics), Elecsys HBsAg II Quant (Roche Diagnostics) and DiaSorin Liaison XL. All assays provide measurements that correlate well with each other (Burdino et al., 2014; Liao et al., 2015). The Architect assay is capable of quantifying HBsAg concentrations ranging from 0.4 to 250 IU/mL (Deguchi et al., 2004). The range of the Elecsys II and DiaSorin Liaison XL assays are respectively from 0.05 to 130 IU/mL (sensitivity from 0.017 to 0.022 IU/mL) and 0.03 to 150 IU/mL (sensitivity of 0.03 IU/mL) (Burdino et al., 2014; Cornberg et al., 2017). All three assays have automatic dilution (1:400) to increase the upper limit of detection to over 50,000 IU/mL.

Unfortunately, these assays do not distinguish between the three forms of HBsAg (small, medium, and large). In pre-clinical trials, the ratios and composition of the three HBsAg forms have been reported to predict HBsAg clearance during treatment in patients with HBeAg-positive CHB (Pfefferkorn et al., 2021). Therefore, the quantification of HBsAg variants and monitoring the HBsAg composition throughout treatment could be important to predict the clearance of secreted HBsAg and the associated reduction in HCC risk.

#### Molecular Association With HCC

Several clinical studies recently reported that high levels of serum HBsAg are associated with an increased risk of HCC (Tseng et al., 2012; Kawanaka et al., 2014). Similarly, HBsAg loss is associated with very low HCC risk (Yip et al., 2017, 2019). While the underlying mechanism is not clear, this may be due to the association of HBsAg with replication levels or the amount of integrated HBV DNA, which are both risk factors for HCC (Xiangji et al., 2011; Yan et al., 2015; Tseng et al., 2019).

Chronic inflammation driven by anti-HBs responses could promote oncogenesis. A chimeric HBV-HCC mouse model was studied by extracting HBsAg-expressing hepatocytes from HBsAg transgenic mice (C57BL/6J) and transferring them into immuno-competent Fah^–/–^ recipient mice (which allow implantation of hepatocytes) (Hao et al., 2021). Persistent HBsAg expression triggered HBsAg-specific CD8+ T cell activation, followed by hepatocyte apoptosis and turnover, progressive chronic inflammation, clonal expansion, and ultimately HCC (Nakamoto et al., 1998; Hao et al., 2021). In *in vitro* models, HBsAg has been reported to promote HCC invasion through the TLR2/MyD88/NF-kB signaling pathway (Cheng et al., 2017).

Hepatitis B surface antigens with mutations in the Pre-S1 or Pre-S2 regions could be directly oncogenic: these mutated proteins can alter host cell lipid metabolism, lead to ER stress, induce oxidative DNA damage and genomic instability, all of which increase the risk of HCC development (Hsieh et al., 2004; Wang et al., 2006; Yang et al., 2008). HBsAg Pre-S1 and Pre-S2 mutants accumulate intracellularly, forming the characteristic cytopathic effect of ground-glass structures (Roingeard and Sureau, 1998). Ground-glass hepatocytes (GGH) occur as either Type I or Type II GGH containing LHBsAg with mutations in the Pre-S1 or Pre-S2 regions, respectively (Wang et al., 2003). Type I GGH occurs as single hepatocytes during early stages of HBV infection with active HBV replication, while Type II GGH occurs as clusters (suggestive of clonal expansion) during latter stages of reduced HBV replication (Fan et al., 2001; Wang et al., 2003). Type II GGH is associated with cirrhosis and HCC development (Fan et al., 2001). In HBV patients, biopsies of cirrhotic nodules with Type II GGH contained HBV genomes which were integrated and clonally expanded, suggesting that Type II GGH are pre-neoplastic lesions (Fan et al., 2000). Similarly, the presence of Pre-S mutants in the serum of patients with CHB is associated with an increased risk of HCC, with Pre-S1 and Pre-S2 mutants present significantly higher in HCC patients compared to non-HCC carriers (19/64, 29.7% vs 25/202, 12.4%, *P* = 0.002) (Fan et al., 2001; Lin C.L. et al., 2007). HBsAg mutants may activate ER stress-dependent and -independent pathways to promote genomic instability and cell proliferation needed for HCC tumorigenesis.

#### Performance as a Predictor of HCC

##### HCC occurrence

Generally, the predictive value of HBsAg levels for HCC is poorer than HBV DNA or ALT in treatment-naïve patients with CHB (Tseng et al., 2012). In HBeAg-negative patients with HBV DNA levels ≥2,000 IU/mL, HBsAg levels (<1,000 IU/mL or >1,000 IU/mL) were not related to the risk of HCC (AUROC 0.58; *P* = 0.247) (Tseng et al., 2012). However, multivariate analysis showed that HBsAg ≥1,000 IU/mL was an independent risk factor for HCC development in HBeAg-negative patients with HBV DNA level <2,000 IU/mL (HR 13.7, 95% CI: 4.8–39.3) (Tseng et al., 2012).

The true sensitivity and specificity of HBsAg in predicting HCC in these patients on NA therapy remains unknown and requires larger cohorts. Furthermore, HCC can still occur in patients with CHB who have undergone HBsAg loss (Chen et al., 2016). In one retrospective study, the annual incidence of HCC after HBsAg seroconversion was 2.85 and 0.29% in cirrhotic and non-cirrhotic patients, respectively (Kim et al., 2015). The risk factors associated with HCC development post-HBsAg seroconversion are: age above 50 (HR: 12.14; 95% CI: 1.61–91.68), male gender (HR: 8.96; 95% CI: 1.17–68.80), and infection with HBV genotype C (Kim et al., 2015). Given that HCC can occur in patients with CHB following HBsAg seroconversion, quantitative HBsAg is unlikely to be a suitable standalone biomarker for HCC risk (Kim et al., 2015; Chen et al., 2016).

##### HCC recurrence

HBsAg can also be used to predict HCC recurrence following curative hepatic resection (HR 1.23, 95% CI: 1.04–1.44, *P* = 0.01) (Huang et al., 2014; Zhou et al., 2015). Moreover, HBsAg level ≥4,000 IU/mL (HR 2.80; *P* = 0.023) is a risk factor for late HCC recurrence (after 2 years) (Sohn et al., 2014). Following hepatic resection, in HBeAg-negative patients with HBV DNA level <2,000 IU/mL, HBsAg level determined the risk of HCC recurrence (*P* = 0.014), while HBV DNA (*P* = 0.55) and ALT (*P* = 0.186) were not predictive (Huang et al., 2014). Univariate analyses of patients with HBV-HCC following radiofrequency ablation showed HCC recurrence is associated with HBsAg ≥1,000 IU/mL (Zhang et al., 2017). Similarly, in HBeAg-negative patients following radiofrequency ablation, recurrence-free survival significantly decreased (*P* = 0.039) as a result of high HBsAg levels; i.e., at 2 years post-treatment, recurrence-free survival decreased from 64% (HBsAg < 1,000 IU/mL) to 50% (HBsAg ≥ 1,000 IU/mL) (Zhang et al., 2017). In summary, high HBsAg may be useful in predicting HCC development and recurrence in HBeAg-seronegative patients with low HBV DNA.

### Hepatitis B Core Related Antigen

#### Description

The 3.5-kb precore RNA derived from the HBV Pre-C/C gene can act as the template for three viral proteins: HBcAg, HBeAg and a truncated 22 kDa precore protein (p22Cr) (Mak et al., 2018). The so-called hepatitis B core related antigen (HBcrAg) consists of these three proteins which share an identical 149 long amino acid sequence. HBcAg forms the viral capsid subunits. HBeAg (164-amino acid protein) is synthesized by removing the C-terminal region of p22 and is secreted from infected cells (Messageot et al., 2003). p22Cr is the pre-core protein with additional post-translational processing at both the N- and C-termini (Kimura et al., 2005).

#### Quantification

Hepatitis B core related antigen was first measured by a sensitive enzyme immunoassay that denatures antibodies prior to analysis and therefore can detect HBcAg and HBeAg in anti-HBc or anti-HBe antibody-positive patients (Kimura et al., 2002). Currently, a newly chemiluminescence enzyme immunoassay with monoclonal antibodies to HBeAg and HBcAg was developed for the detection of HBcrAg. This assay showed the HBcrAg concentration correlates strongly with the HBV DNA concentration (*P* < 0.001) over a 5-log range. Moreover, the correlation between HBV load and circulating HBcrAg was not affected in HBeAg-negative patients nor those with pre-core mutations (Rokuhara et al., 2003). Particularly for patients under NA treatment, the HBcrAg assay could be a sensitive and clinically useful surrogate marker of intrahepatic HBV cccDNA levels (Rokuhara et al., 2003).

#### Molecular Association With HCC

Transcriptional activity of intrahepatic cccDNA is recognized as a risk for HBV-induced HCC under NA therapy (Levrero and Zucman-Rossi, 2016; Chen et al., 2017; Mak et al., 2018; Suzuki et al., 2019; Testoni et al., 2019). Several studies have shown that serum HBcrAg is highly correlated with intrahepatic cccDNA activity (Wong et al., 2017; Mak et al., 2018) as it can only be expressed from cccDNA (unlike HBsAg, which can also be expressed from integrated HBV DNA). Importantly, NA therapy only inhibits reverse transcription of HBV RNA, but does not inhibit protein synthesis from cccDNA (Tong and Revill, 2016). Therefore, HBcrAg is a non-invasive biomarker of active viral replication, which in turn may predict HCC.

#### Performance as a Predictor of HCC

##### HCC occurrence

A number of studies have suggested that serum HBcrAg can be a useful viral biomarker for HCC risk (Chen et al., 2018; Hosaka et al., 2019; Suzuki et al., 2019; Tseng et al., 2019; Baudi et al., 2020). A study of 1,031 NA-naïve patients with CHB (78 of whom were diagnosed with HCC during the follow-up period, median duration 10.7 years) revealed that serum HBcrAg was significantly related to the risk of developing HCC. HBcrAg >794 U/mL (HR, 5.05; 95% CI, 2.40–10.63) was associated with the risk of developing HCC, independent of HBV DNA titers. In the subgroup of HBeAg-negative, non-cirrhotic patients with HBV DNA levels ≤10,000 copies/mL, HBcrAg >5,012 U/mL was significantly related to the risk of HCC (HR 6.13, 95% CI 1.71–22.06). However, in the subgroup of CHB patients with HBV DNA levels >10,000 copies/mL, any HBeAg status, and FIB-4 index ≤3.6 (an index of fibrosis), HBcrAg >794 U/mL was associated with the incidence of HCC (HR 5.69, 95% CI 1.37–23.72) (Tada et al., 2016). Another study of 2,666 patients with CHB (of whom 209 developed HCC) reported that baseline HBcrAg levels of >10 kU/mL in HBeAg-negative patients with HBV DNA levels from 2,000 to 19,999 IU/mL are at increased risk of HCC (Tseng et al., 2019). Conversely lower HBcrAg levels (<10 kU/mL) were linked to a low risk of HCC.

Hepatitis B core related antigen has been reported to be superior to HBV DNA or HBsAg in predicting HCC in NA-naïve patients with CHB. Tada et al. (2016) reported that HBcrAg could predict HCC in 2, 5, and 10 years with AUROC curves 0.80, 0.68, 0.70 (compared to HBV DNA at 0.75, 0.63, 0.65, respectively). Moreover, Tseng et al. (2019) found that AUROC of HBcrAg, HBV DNA, HBsAg was 0.73, 0.72, 0.57, respectively with 10 years follow-up, or 0.70, 0.69, 0.56 with 15 years follow-up. These studies show a high correlation between HBV DNA and HBcrAg levels, with HBcrAg being more sensitive than HBsAg in predicting HCC in untreated patients.

In patients with CHB under NA treatment, persistently high HBcrAg levels were associated with HCC development (Kumada et al., 2013; Hosaka et al., 2019). Hosaka et al. (2019) reported that in a study of 1,268 patients treated with NAs for more than 1 year, among the 60 of 667 HBeAg-positive patients, high HBcrAg levels (≥4.9 log U/mL) after 1-year on-treatment was associated with increased HCC incidence within 15 years (HR, 6.15, 95% CI: 1.89–20.0, *P* = 0.003). Using a HBcrAg cut-off value of 4.9 log U/mL gave positive and negative predictive values of 0.95 and 0.19, with sensitivities and specificities of 0.903 and 0.217, respectively. Moreover, in 601 HBeAg-negative patients, the risk of HCC was higher in those with HBcrAg values >4.4 log U/mL (HR, 2.54, 95% CI: 1.40–4.60; *P* = 0.002). In this cohort positive and negative predictive values were 0.51 and 0.79, sensitivity and specificity were 0.519 and 0.787 (Hosaka et al., 2019). This result is similar to another study enrolling 76 NA-treated patients with CHB with undetectable HBV DNA diagnosed with HCC and 152 matched controls who did not develop HCC (Cheung et al., 2017). The AUROC of HBcrAg in the HCC group was 0.61 (95% CI: 0.54–0.69) for predicting HCC. Using a cut-off value of ≥7.8 kU/mL, the sensitivity, specificity, positive predictive value (PPV) and negative predictive value (NPV) were 57.9, 70.4, 49.4, and 77.0%, respectively, with an odds ratio (OR) of 3.27 (95% CI: 1.84–5.80) for HCC development. HBcrAg was more predictive of HCC in non-cirrhotic patients: AUROC was 0.70 (95% CI: 0.58–0.81) using a HBcrAg cut-off value of ≥7.9 kU/mL, with a sensitivity, specificity, PPV and NPV of 62.5, 78.1, 58.8, 80.6%, respectively, and with an OR of 5.95 (95% CI: 2.35–15.07) for HCC development (Cheung et al., 2017).

##### HCC recurrence

Hepatocellular carcinoma recurrence after HCC resection is still high, with a rate of ∼50% within two years (Wu et al., 2009). High serum HBcrAg has been reported to predict HCC recurrence: Chen et al. (2018) reported that in 56 of 89 HCC patients with both positive cccDNA and HBcrAg who had been followed up for 5 years, recurrence rates of HCC in patients with high HBcrAg (>5.2 log U/mL) were higher than those with low HBcrAg (≤5.2 log U/mL; *P* = 0.003).

During NA therapy, higher HBcrAg levels at HCC diagnosis can predict post-treatment recurrence of HCC (Hosaka et al., 2010). In a study of 55 HCC patients with NA treatment at diagnosis of HCC receiving curative surgery, serum HBcrAg levels ≥4.8 log U/mL at the time of HCC diagnosis was an independent risk factor for HCC recurrence with HR of 8.96 (95% CI: 1.94–41.4) (Hosaka et al., 2010). A long-term follow-up study in Netherlands revealed that higher HBcrAg level (≥5.1 log U/mL) was associated with an increased tumor recurrence rate in 53 of 119 HCC patients who were identified with early stage HCC receiving NAs at the time of HCC diagnosis (Beudeker et al., 2021). Moreover, in a cohort of 357 CHB-related HCC patients who underwent liver transplantation followed by NA treatment, HBcrAg ≥5.0 log U/mL was an independent risk factor for HCC recurrence, with a higher 5-year cumulative recurrence rate, compared with an HBcrAg <5.0 log U/mL (37.6 vs 6%, *P* < 0.001) (HR:5.27, 95% CI 2.47–11.25, *P* < 0.001) (Yu et al., 2019). In conclusion, HBcrAg may be a useful biomarker for HCC recurrence, however the sensitivity and specificity of HBcrAg in predicting HCC recurrence needs further research.

## Future Work and Conclusion

This review has assessed the value of serum viral biomarkers in HBV-related HCC. Of all the potential biomarkers that have been studied, growing evidence supports the use of serum HBcrAg and preS mutations as biomarkers for predicting HCC occurrence in people with CHB, both in NA-naïve patients and in patients receiving NA treatment. In combination with AFP and abdominal ultrasound serum biomarkers might improve HCC screening and increase early diagnosis, although further validation studies are required to confirm their clinical performance in predicting and/or detecting HCC. Moreover, several biomarkers remain to be tested in a clinical setting (e.g., HBV integrations and HBV RNA, both full length and truncated forms), laying the groundwork for future exploratory studies.

Challenges remain in this field of research. Firstly, some of these markers (e.g., HBV RNA and HBcrAg) have no standardized quantification assay. To accurately and robustly compare HCC risk between different studies, equivalent cut-off values need to be used and this can only be done with appropriate reference samples and standardized assays. Moreover, we lack the appropriate laboratory models to investigate new and existing HCC markers in HBV infection. Even if there were a practical experimental animal system that supported HBV infection, no known models recapitulate the decades long-process of HBV-initiated HCC. This makes discovery, characterization, and confirmation of new and existing viral biomarkers difficult.

While this field awaits further developments to enable more in-depth analysis, our review has shown signs of promise in viral biomarkers and their ability to predict HBV-associated HCC occurrence and recurrence. We expect that (in combination with existing markers) viral biomarkers will increasingly become incorporated into HCC risk algorithms, improving health outcomes for the ∼300 million people worldwide living with CHB.

## Author Contributions

YL initiated the writing, led the organization, and did the majority of the research for the review. VV and MP produced the figure and contributed to large sections of the review. JG, JF, and MD were involved in editing the manuscript and provided crucial input into several sections of the review. TT was responsible for the conceptualization, coordination, structure, and editing of the review. All authors contributed to the article and approved the submitted version.

## Conflict of Interest

The authors declare that the research was conducted in the absence of any commercial or financial relationships that could be construed as a potential conflict of interest.
